# A novel bamboo sheet chair and its influence on sitting comfort

**DOI:** 10.7717/peerj.9476

**Published:** 2020-07-01

**Authors:** Fangcheng Yuan, Yong Guo, Yunjiao Shi, Kaiting Zhang, Zhenzhen Zhu, Yuxia Chen

**Affiliations:** College of Forest and Garden, Anhui Agricultural University, Hefei, China

**Keywords:** Sitting comfort, Pressure distribution, Bamboo sheet chair, Body characteristics

## Abstract

**Background:**

In today’s economy, workers spend increasingly more time in seated positions, leading to a growing scientific interest in chair design. In this study we used body pressure distribution tests to compare a novel bamboo chair with unique structural features to other commonly-used chairs. We studied the bamboo sheet chair’s physical characteristics and comfort to provide a scientific theoretical basis for common use seat design.

**Methods:**

A total of 25 (14 male and 11 female) subjects participated in the study. Subjects were divided into six groups according to their body characteristics parameters included stature, weight, shoulder breadth, hip breadth, waist width, popliteal height, buttocks-popliteal length, and buttock-abdomen depth, with three groups for males and three groups for females. Each subject was required to complete specified body pressure tests for three different experimental chairs for three minutes and subjective comfort evaluations were also administered. The pressure indexes were measured from the seat pan and backrest and calculated with MATLAB 2015b, which mainly included maximum pressure (*Pm*), average pressure (*Pa*), pressure exponent (*Pe*) and contact area index (*P*_*AI*_). Three pressure threshold limits of 0.67 kPa, 4.00 kPa and 9.33 kPa and four contact surface indexes were used in the experiment to reflect the contact area between human and chair.

**Results:**

The contact areas in the backrest (52.96 ± 32.94 cm^2^) and seat pan (307.75 ± 90.31 cm^2^) in the middle-to-high threshold pressure range, and the contact areas of the backrest (4.34 ± 5.95 cm^2^) in the high threshold pressure range of bamboo sheet chair were smaller than the corresponding indexes of the common office chair (81.430 ± 45.04 cm^2^, *p* = 0.00; 394.39 ± 98.99 cm^2^, *p* = 0.02; 13.54 ± 12.00 cm^2^, *p* = 0.00, respectively). The pressure index (2.68 ± 0.88 kPa), maximum pressure (6.66 ± 2.05 kPa), and average pressure (2.42 ± 0.59 kPa) values of the bamboo sheet chair backrest were also found to be lower than those of the office chair (4.32 ± 1.62 kPa, *p* = 0.00; 10.50 ± 3.88 kPa, *p* = 0.00; 3.43 ± 0.97 kPa, *p* = 0.00, respectively). The average pressure on the seat pan was greater than 4 kPa for all subjects, while the average pressure on the seat pan was greater than 9.33 kPa for male subjects with a body mass index (BMI) of 27.48.

**Discussion:**

The bamboo sheet chair’s contact areas within the middle-to-high and high-pressure threshold ranges of the backrest and seat pan were smaller than those of the office chair, indicating that the bamboo sheet chair is effective at relieving pressure. Human body characteristics must be considered in the design of seat functional size. Buttocks-popliteal length, weight, body mass index, body shape and weight distribution, all have important effects on the distribution of body pressure at the human-chair interface.

## Introduction

The chair is an indispensable piece of furniture for seated activities. It is the most frequently used piece of furniture and is customized for human use (Ma, Tian & Sun, 2010). The ultimate goal of chair design is to help people live a more comfortable, healthy, and refined lifestyle (Dainoff et al., 2007). Future chair designs should consider the properties of raw materials and the structural model to better follow ergonomic guidelines (Huang & Liu, 2013). Work environments in today’s society are fast paced and this pace has increased the amount of time office workers spend in seated positions (Aota et al., 2007). Research has found an adverse relationship between sedentary behavior and health (Owen et al., 2011; Salmon et al., 2011). Workers who sit for too long often report discomfort, including leg numbness, back pain, or the inability to stand straight (Lynch & Neville, 2015; Vollowitz, 1988). There are two main factors affecting sitting comfort: not recommended seating position, e.g., an excessive forward lean; chair design flaw, e.g., the sitting depth is too large, or if the backrest angle is too small (Keegan, 1953). Furthermore, sitting discomfort often leads to musculoskeletal injuries (Vink & Hallbeck, 2012) involving the muscle, nerve, tendon, ligament, joint, cartilage, or intervertebral disc (Collins & Raleigh, 2009). Chair quality largely determines sitting comfort. Chairs are mainly constructed out of wood, metal, plastic, and composite materials (Zhang & Zhang, 2011). Among these materials, wooden and metallic chairs are often too hard for worker, and plastic has poor durability (Blaga, 1984). Additionally, plastic and foaming composite materials are petrochemical products, which are harmful to both human health and the environment (North & Halden, 2013). Therefore, exploring alternatives to non-renewable resources and developing environment-friendly furniture are necessary for energy saving, emissions reduction and developing a low-carbon economy. As health and environmental awareness increase, consumers are demanding more environmentally-friendly furniture (Chang & Hsieh, 2016). Natural wood has traditionally been the raw material used for furniture manufacturing. China’s timber demand has been greater than its supply (Zhang & Feng, 2015). This supply–demand imbalance is quite high, especially for wood used for furniture. It is imperative to use high-quality ecological materials with short growth cycles instead of wood (Cristóbal, 2007).

Bamboo is eco-friendly, making it a good option for home furnishing (Wang & Tian, 2015). There are 1,250 species, and 75 genera, of bamboo, most of which are relatively fast-growing (Scurlock, Dayton & Hames, 2000). In China, there are about 500 bamboo species with 40 genera (Yang & Hui, 2010). Bamboo reproduces quickly, grows fast, matures early, and has high yields (Hakeem et al., 2015). Moreover, bamboo’s advantages over wood and steel lie in its strength, elasticity, reliability, low density, higher specific strength, high thermal conductivity, and specific stiffness (Osorio et al., 2010; Li et al., 2010). Bamboo’s durability is higher than wood and its growth cycle is also much shorter (Flander & Rovers, 2009).

In this study, a manufactured novel bamboo sheet chair made use of the favorable mechanical properties of natural bamboo materials through its processing and design. We then compared our chair with two other common chairs and explored the influence of body characteristics on sitting comfort. A previous study (Zhang, Helander & Drury, 1996) identified multidimensional properties of comfort and discomfort. Feelings of discomfort were associated with pain, tiredness, soreness and numbness (Helander & Zhang, 1997). Kamp (2012) used subjective and objective evaluations to study the influence of car seat design on user experience. Bi et al. (2013) looked at how posture affected long-term comfort in office chairs. Samuelsson et al. (2009) studied the effect of shaped wheelchair cushions and lumbar supports on under-seat pressure, comfort, and pelvic rotation. Although these studies have thoroughly identified the properties and factors of comfort and discomfort, they have not used human body pressure indicators combined with subjective comfort evaluations to characterize chair comfort. It is worth mentioning that previous studies on human body pressure have been mostly concerned with mattresses (Li, Shen & Zhou, 2009; Miguel et al., 2008), sofas (Chen, Shen & Guo, 2010; Hu et al., 2017), and non-wooden chairs (Bui et al., 2017; Liu & Griffin, 2018). Vlaović et al. (2012) reported on feelings of comfort while sitting on four different office chairs with various types of upholstered seats, and compared them to objective indicators, such as sex, mass, height, and body mass index (BMI). They found that pressure rose with an increase in BMI for men but had the opposite effect for women. Their study analyzed the influence of sex, mass, height, and BMI on pressure and comfort while sitting in different office chairs, but specific body characteristics such as shoulder and waist width were not taken into account. Only a few studies have focused on wooden chairs. Zhong & Sun (2014) focused on analyzing wooden chair production processes and equipment. Horman et al. (2010) presented a numerical analysis of the stress and strain conditions of a three-dimensional wooden chair skeleton. Uysal, Haviarova & Eckelman (2015) studied the cyclic durability, ease of disassembly, repair, and reuse of wooden chair frames. These studies did not use body pressure monitoring to objectively evaluate wooden chair comfort, and they did not discuss the effect of physical characteristics on sitting comfort.

We used body pressure distribution testing on different body types to study the bamboo sheet chair’s comfort, and the objective data was combined with subjective evaluation. The results were compared to those of two commonly used chairs to determine whether the bamboo sheet chair has a better sitting comfort.

## Materials and Methods

### Participants

Twenty-five participants (14 males and 11 females) volunteered for this study. The overall mean ± SD of male participants for age, mass and stature were 21.71  ± 1.98 years, 66.49  ± 8.23 kg and 173.00  ± 4.74 cm, respectively. Female participants’ mean age, mass and stature were 21.36  ± 2.01 years, 53.83  ± 4.07 kg and 161.74  ± 3.73 cm, respectively. We divided the 14 male subjects into three groups and the 11 female subjects into three groups according to their body characteristics using IBM SPSS statistics software (version 20.0; IBM Corp., USA) to conduct cluster analysis.

All the participants were healthy with no musculoskeletal injuries in the past 12 months. The participants were asked to refrain from strenuous exercise, caffeine and alcohol in the 24 h period prior to each testing session. All participants provided consent in compliance with the Declaration of Helsinki (ver. 7.0; October 2013) (World Medical Association, 2013). All protocols were approved by the Anhui Agricultural University’s ethical committee.

### Manufacturing

**Figure 1:**
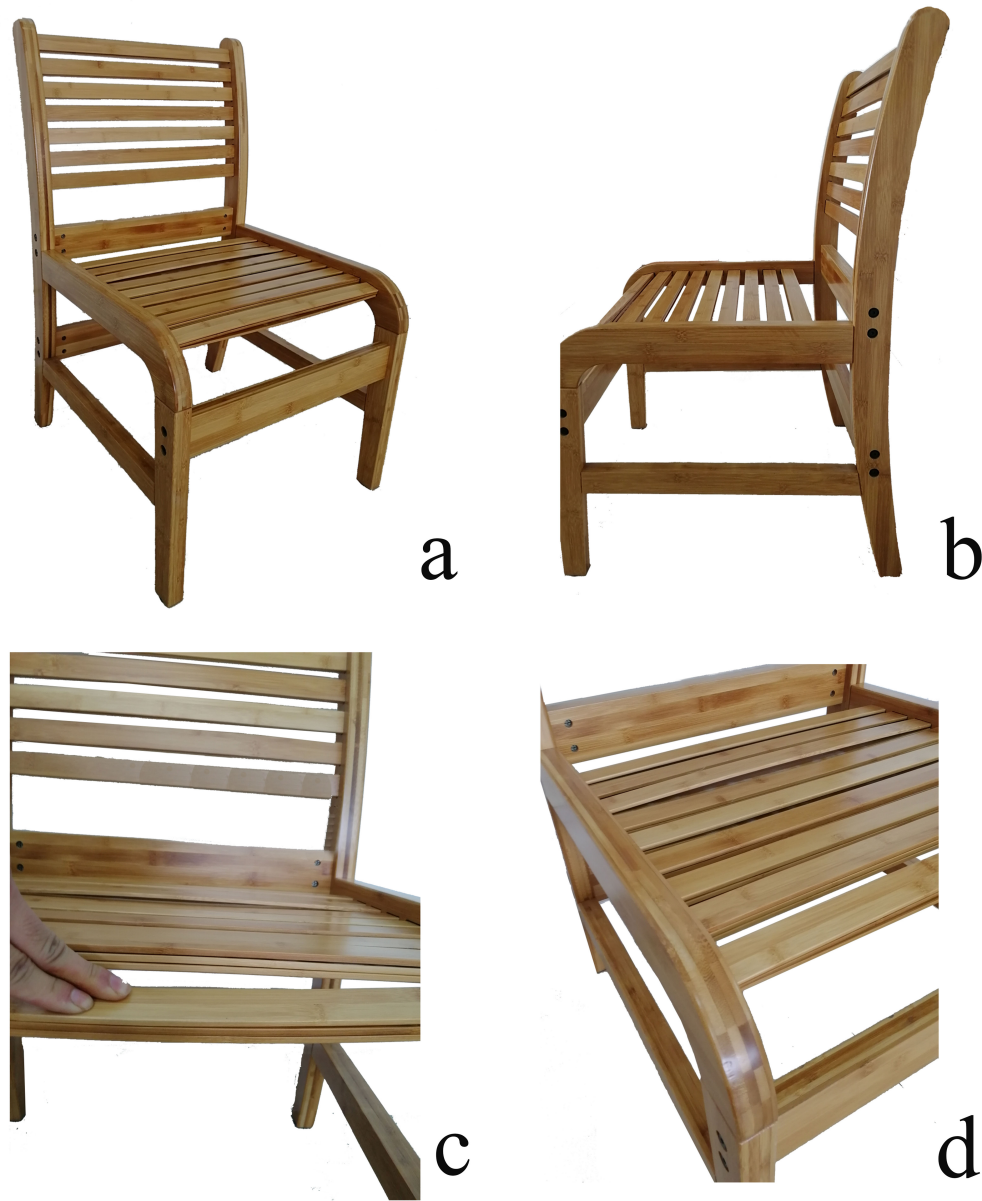
Structure of the novel bamboo sheet chair (A-D)

A novel sheet chair was constructed from natural bamboo. First, we cut the rectangular bamboo sheets with a thickness of 5 mm. These sheets underwent six cycles of anti-corrosion, anti-insect, and anti-moth processing, as well as drying, carbonization, and assembly surface decoration. According to the requirements, the seat pan and backrest of the chair were stacked with three layers of bamboo sheets, and the bamboo sheets were fixed using slots. The bamboo sheets in each layer could be moved up and down for vertical elastic support. The structural design is shown in Fig. 1.

### Experimental design and procedure

The office chairs (Model:KL-702) used in the experiment were purchased from Anhui JinRuiXin Office Furniture Co. Ltd., China. We designed both the bamboo sheet chairs and wooden chairs, which were commissioned by the cooperative enterprise, Anhui Longhua Bamboo Product Co. Ltd., China.

**Figure 2:**
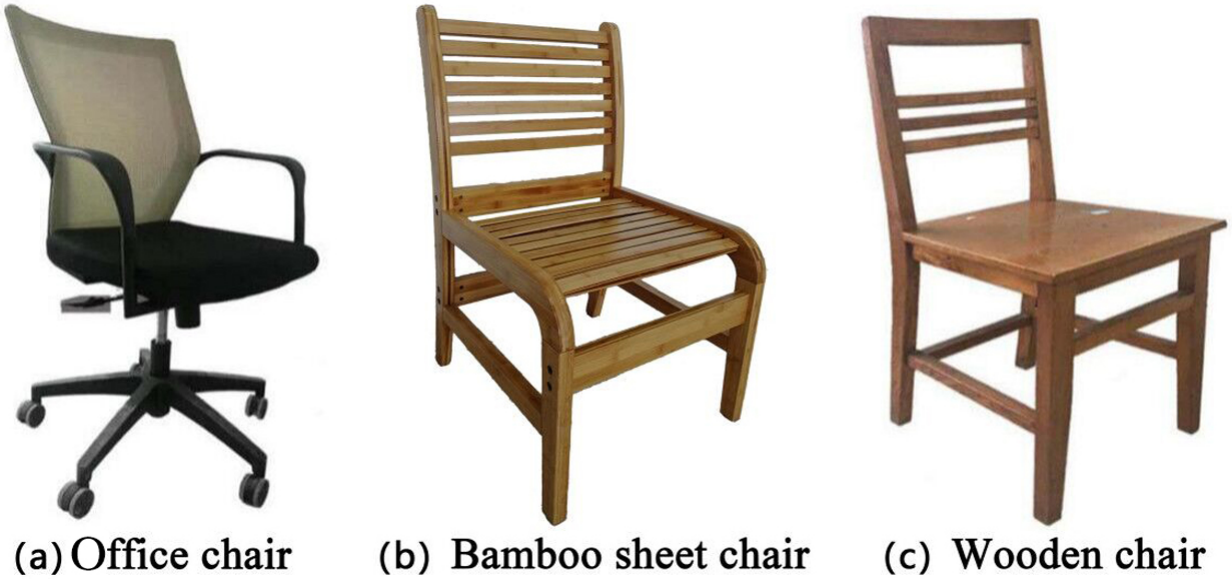
The three experimental chairs. The study evaluated three chairs: an office chair (A), a self-made bamboo sheet chair (B), and a wooden chair (C).

**Figure 3:**
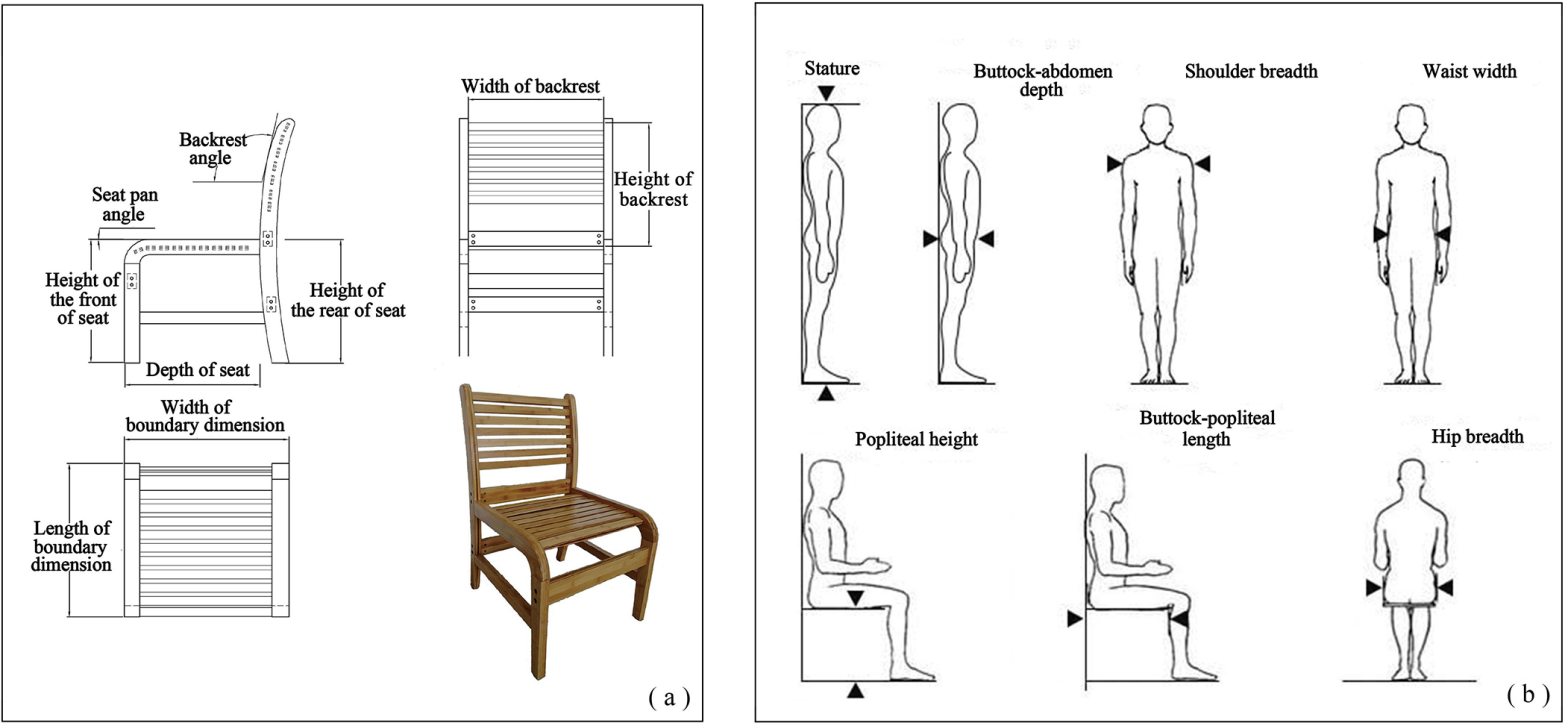
(A) The measurement indices of chairs; (B) detailed measurement methods of body characteristics (Pheasant, 2005).

The study evaluated three chairs: an office chair, a self-made bamboo sheet chair, and a wooden chair (Fig. 2). The dimensions of the three chairs are listed in Table 1, and the measurement indices are shown in Fig. 3A. We measured body characteristic parameters included stature, weight, shoulder breadth, hip breadth, waist width, popliteal height, buttocks-popliteal length, and buttock-abdomen depth. Detailed measurement methods are shown in Fig. 3B (Pheasant, 2005). We divided the male subjects into three groups and the female subjects into three groups according to their body characteristics using cluster analysis in the IBM SPSS statistics software (version 20.0; IBM Corp., USA).

Before reporting comfort scores on the “Subjective evaluation for sitting comfort of seven-degree scale” questionnaire, the subjects were not informed of the objective of the test. The subjective sitting comfort evaluation was conducted by combining the semantic differentiation method with the free modulus amplitude estimation method (Zhang, Jiang & Wang, 2004). Semantic differentiation is expressed in the form of a seven-level scale that mainly evaluates the comfort of various body parts (Guo, Sheng & Chen, 2011). The subjective method was based on the participants’ feelings of comfort or discomfort while sitting on chairs during a given period of time Subjects could freely adjust their sitting positions to determine whether they are comfortable or to identify discomfort in different seats for comfort comparison. The questionnaire consisted of seven rating categories that asked the participants to evaluate the intensity of pressure and level of comfort or discomfort while sitting. Corresponding numerical values were then circled by the participants. The presented categories are listed in Table 2. The chairs’ identities were deliberately hidden, so that the participants could not be influenced by any predisposed bias/opinion. Although the chairs were covered, the office chair’s arm rests were an identifiable feature; this is a shortcoming of the study design.

**Table 1:** Size parameters of the experimental chair (Mean + SD).

Type	Depth of seat/cm	Height of the front of seat/ cm	Height of the rear of seat/ cm	Seat width/ cm	Height of backrest/ cm	Width of backrest/ cm	Boundary dimension/ cm	Backrest angle/ °	Seat pan angle/ °
Office chair	46.1	47.1	46.3	45.8	42.7	45.7	55.4 × 45.8 × 89	117.70	1.21
Bamboo sheet chair	46	43.6	41.8	46.8	42	46.7	58.3 × 52.9 × 87	104.12	2.24
Wooden chair	36.4	45	44.1	40.5	41.5	37	39.4 × 40.5 × 85	97.97	1.42

**Table 2:** Seven-degree scale on semantic differential methods. The questionnaire consisted of seven rating categories that asked the participants to evaluate the intensity of pressure and level of comfort or discomfort while sitting. Corresponding numerical values were then circled by the participants.

Type/Seven-degree scale		**1**	**2**	**3**	**4**	**5**	**6**	**7**
Softness		Very soft	Soft	A little bit soft	Medium	A little bit hard	Hard	Very hard
Stability		Very stable	Stable	A little bit stable	Medium	A little bit unstable	Unstable	Very unstable
The comfort of thigh and buttock	The front of thigh	Very comfortable	Comfortable	A little bit comfortable	Medium	A little bit sore	Sore	Very sore
	The backend of thigh	Very comfortable	Comfortable	A little bit comfortable	Medium	A little bit sore	Sore	Very sore
	Shank	Very comfortable	Comfortable	A little bit comfortable	Medium	A little bit sore	Sore	Very sore
	Caudal vertebrae	Very comfortable	Comfortable	A little bit comfortable	Medium	A little bit sore	Sore	Very sore
	Ischium	Very comfortable	Comfortable	A little bit comfortable	Medium	A little bit sore	Sore	Very sore
Thigh pressure	Inner	No compression	Low compression	Middle-to-high compression	High compression	*	*	*
	Lateral	No compression	Low compression	Middle-to-high compression	High compression	*	*	*
Elasticity of seat		Very elastic	Elastic	Medium	A little bit elastic	Inelastic	*	*
General sensation		Very comfortable	Comfortable	A little bit comfortable	Medium	A little bit uncomfortable	Uncomfortable	Very uncomfortable

Each subject completed a body pressure test for each of the three different chairs. During the test, the participants were asked to naturally depend on the backrest, and keep their eyes straight ahead, thighs were roughly parallel, knees bent at approximately 90°, feet flat on the floor, shoulders relaxed, and hands gently resting on the thighs (Chen et al., 2009). The CONFOR Mat Pressure Measurement System (BPMS, 5400D, Tekscan Inc., USA) was used to evaluate the pressure distribution between the chair and the subject. During the test, the pressure sensing mat was placed between the subject, seat pan and backrest. After the pressure distribution became relatively stable, the data was recorded for 3 min with a sampling rate of 8 f s^−1^. The subjects were required to sit in the test chair according to the specified sitting posture during the duration of the body pressure test. Participants were not allowed to talk during the body pressure test.

### Data processing and reduction

Pressure data were converted into a pressure distribution matrix ASCII file. Considering the pressure matrix as the basis of calculations and statistical analysis, the pressure distribution index values for the seat pan and backrest were calculated using MATLAB 2015b, which mainly included maximum pressure (*P*_*m*_), average pressure (*P*_*a*_), pressure exponent (*P*_*e*_) and contact area index (*P*_*AI*_).

(1) Maximum pressure (*P*_*m*_)

The maximum pressure was defined as the pressure at the 95th percentile of all test points, and is described in Eq. (1).

(1) \begin{eqnarray*}& & {P}_{m}={\overline{P}}_{N}+ \left( SD\times K \right) \end{eqnarray*}

where *N* is the number of measurement points, ${\overline{P}}_{N}$ isthe average pressure of all the measurement points, *SD* is the standard deviation of all test points, and *K* = 1.64. Furthermore, ${\overline{P}}_{N}$ and *SD* are outlined Equations in (2) and (3), respectively.

(2) \begin{eqnarray*}{\overline{P}}_{N}& = \frac{1}{N} \sum _{i=1}^{N}{P}_{i}\end{eqnarray*}

(3) \begin{eqnarray*}SD& =\sqrt{ \frac{1}{N-1} \left( \sum _{i=1}^{N}{P}_{i}^{2}-N{\overline{P}}_{N}^{2} \right) }\end{eqnarray*}

where *N* is the number of test points, and *P*_*i*_ is the pressure of each test point.

(2) Average pressure (*P*_*a*_)

Average pressure was defined as the 50th percentile pressure value of all test points, which is the arithmetic mean of all test points, and is given by Eq. (4).

(4) \begin{eqnarray*}{P}_{a}= \frac{1}{N} \sum _{i=1}^{N}{P}_{i}\end{eqnarray*}

where *N* is the number of test points, and *P*_*i*_ is the pressure of each test point.

(3) Pressure exponent (*P*_*e*_)

Pressure exponent was calculated using Eq. (5).

(5) \begin{eqnarray*}{P}_{e}=\sqrt{{ \left( {P}_{M10}-10 \right) }^{2}+S{D}_{10}^{2}}\end{eqnarray*}

where *P*_*M*10_ is the average value of the contact surface pressure greater than 1.33 kPa, and *SD*_10_ is the standard deviation of the contact surface pressure greater than 1.33 kPa (Shelton, Barnett & Meyer, 1999). Additionally, *P*_*M*10_ and *SD*_10_ are described by Eqs. (6) and (7), respectively.

(6) \begin{eqnarray*}{P}_{M10}& = \frac{1}{N} \sum _{i=1}^{{N}_{10}}{P}_{i}\end{eqnarray*}

(7) \begin{eqnarray*}S{D}_{10}& =\sqrt{ \frac{1}{{N}_{10}-1} \left( \sum _{i=1}^{{N}_{10}}{P}_{i}^{2}-{N}_{10}{P}_{M10}^{2} \right) }\end{eqnarray*}

where *P*_*i*_ ≥1.33 kPa, and *N*_10_ is the number of pressure test points greater than 1.33 kPa.

(4) Contact area index (*P*_*AI*_)

Contact area index was defined as the “pressure contact area within a certain threshold pressure limit range”. Under normal circumstances, the capillary pressure of the arterioles is 3.3–4.6 kPa, the pressure in the venules is approximately1.6 kPa, the critical pressure is considered to be 4 kPa (Forciea, Humphrey & Qaseem, 2015; Keller, 2006). Therefore, three pressure threshold limits of 0.67 kPa, 4.00 kPa and 9.33 kPa and four contact surface indexes were used in the experiment to reflect the contact area between human and chair. We determined that a human-chair interface pressure between 0.67–4 kPa was a low-pressure threshold value, 4.00–9.33 kPa was a middle-to-high pressure threshold value, and 9.33 kPa and above was a high-pressure threshold value.

### Statistic analysis

Statistical analysis was performed by using IBM SPSS statistics software (version 20.0; IBM Corp., USA). Variance test analysis was applied to the body characteristics we measured, the objective pressure indexes, and the subjective comfort evaluations. The variance of the pairwise comparisons between the various indicators is also listed in Tables 3 and 4.

**Table 3:** The contact area and body pressure distribution parameters on the human-chair interface: backrest/seat.

	Index	Office chair	Bamboo sheet chair	Wooden chair	*F*	Sig
Backrest	St/cm^2^	314.96 ± 118.62^a^	325.55 ± 73.99^a^	182.22 ± 87.52^b^	16.88	0.00
	Sl/cm^2^	192.81 ± 89.49^a^	234.22 ± 55.80^b^	120.58 ± 63.03^c^	15.78	0.00
	Sm/cm^2^	81.430 ± 45.04^a^	52.96 ± 32.94^b^	32.90 ± 26.83^b^	11.17	0.00
	Sh/cm^2^	13.54 ± 12.00^a^	4.34 ± 5.95^b^	3.73 ± 5.67^b^	10.28	0.00
	Pressure exponent/kPa	4.32 ± 1.62^a^	2.68 ± 0.88^b^	2.85 ± 1.16^b^	12.42	0.00
	Maximal pressure/kPa	10.50 ± 3.88^a^	6.66 ± 2.05^b^	6.94 ± 2.58^b^	12.72	0.00
	Average pressure/kPa	3.43 ± 0.97^a^	2.42 ± 0.59^b^	2.53 ± 0.86^b^	10.79	0.00
Seat	St/cm^2^	1111.81 ± 136.75^a^	888.79 ± 124.87^b^	791.56 ± 103.01^c^	43.23	0.00
	Sl/cm^2^	418.79 ± 82.05^a^	324.33 ± 53.45^b^	278.24 ± 57.27^c^	28.72	0.00
	Sm/cm^2^	394.39 ± 98.99^a^	307.75 ± 90.31^b^	270.94 ± 82.61^b^	11.65	0.00
	Sh/cm^2^	268.17 ± 63.39^a^	232.17 ± 75.65^b^	224.15 ± 87.83^b^	2.27	0.00
	Pressure exponent/kPa	9.59 ± 1.94^a^	10.83 ± 1.76^b^	11.92 ± 2.03^b^	8.87	0.00
	Maximal pressure/kPa	23.08 ± 4.82^a^	26.26 ± 4.28^b^	28.91 ± 4.90^c^	9.35	0.00
	Average pressure/kPa	7.19 ± 1.19^a^	7.770 ± 1.30^b^	8.33 ± 1.41^c^	5.15	0.01

**Notes.**

St, Sl, Sm and Sh refer to total contact area of human-chair, contact area in low pressure threshold (from 0.67 kPa to 4.00 kPa), contact area in middle-to-high pressure threshold (from 4.00 kPa to 9.33 kPa) and contact area in high pressure threshold (over 9.33 kPa), respectively.

The different superscript indicates that there is significant difference (*p* < 0.05) between the two types. On the contrary, there is no significant difference.

**Table 4:** The subjective comfort evaluation of three different chairs (Mean + SD + ANOVA).

Type		Office chair	Bamboo sheet chair	Wooden chair	*F*	Sig
Softness		2.04 ± 0.77^a^	3.00 ± 0.75^b^	6.16 ± 0.61^c^	218.28	0.00
Stability		2.04 ± 0.66^a^	1.84 ± 0.73^a^	1.92 ± 0.62^a^	0.53	0.59
The comfort of thigh and buttock	The front of thigh	2.60 ± 0.98^a^	2.56 ± 0.64^a^	3.84 ± 1.00^b^	16.02	0.00
	The backend of thigh	2.16 ± 0.97^a^	2.36 ± 0.74^a^	3.84 ± 0.97^b^	25.06	0.00
	Shank	2.72 ± 0.45^a^	2.56 ± 0.80^a^	3.56 ± 1.13^b^	9.73	0.00
	Caudal vertebrae	2.56 ± 0.64^a^	2.76 ± 0.76^a^	3.64 ± 0.93^b^	12.79	0.00
	Ischium	2.32 ± 0.84^a^	2.68 ± 0.93^a^	3.64 ± 0.97^b^	13.38	0.00
Thigh pressure	Inner	1.80 ± 0.75^a^	1.52 ± 0.57^a^	2.16 ± 0.73^b^	5.20	0.01
	Lateral	1.80 ± 0.57^a^	1.48 ± 0.64^a^	1.88 ± 0.65^a^	2.79	0.07
Elastic of seat		2.76 ± 0.81^a^	2.28 ± 0.66^b^	4.76 ± 0.43^c^	96.84	0.00
General sensation		2.76 ± 0.99^a^	2.20 ± 0.49^b^	4.56 ± 0.64^c^	67.21	0.00

**Notes.**

The smaller the number in the table, the better the indicators.

## Results

### Pressure indexes and subjective comfort evaluation of the three chairs

**Figure 4:**
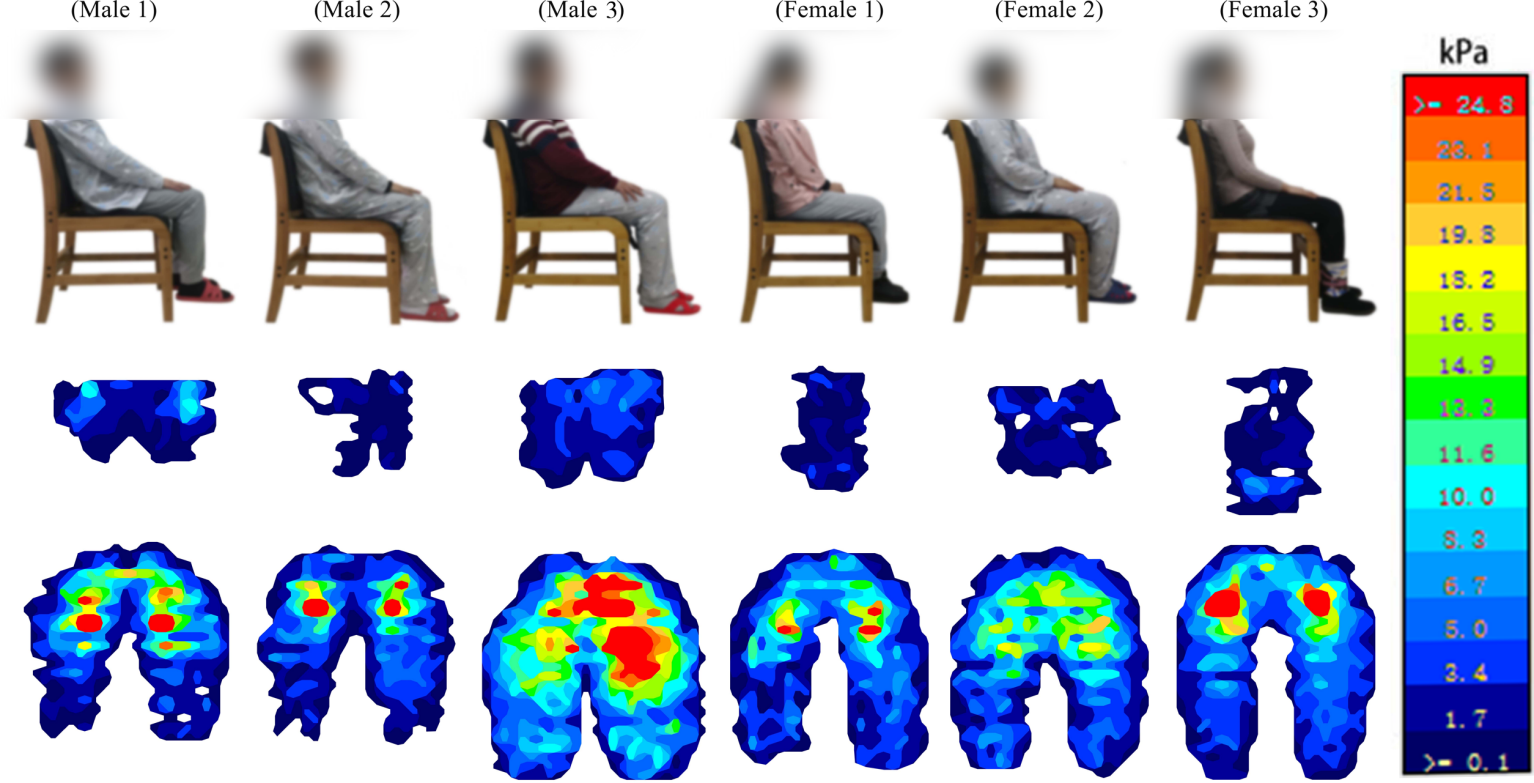
Body pressure distribution of subjects on human-chair interface.

Figure 4 shows a representative body pressure distribution of the human-chair interface. Table 3 lists the three chairs’ seat pan and backrest data, including their contact areas; the contact areas within the low, middle-to-high, and high-pressure threshold ranges; pressure exponents; maximum pressure; and average pressure. The total backrest contact areas of the office chair (314.96 ± 118.62 cm^2^) and bamboo sheet chair (325.55 ± 73.99 cm^2^) were not significantly different, while the backrest contact area of the office chair in the low threshold pressure range (192.81 ± 89.49 cm^2^) was lower than that of the bamboo sheet chair (234.22 ± 55.80 cm^2^, *p* = 0.047). In addition, the contact areas in the backrest (52.96 ± 32.94 cm^2^) and seat pan (307.75 ± 90.31 cm^2^) in the middle-to-high threshold pressure range, and the contact areas of the backrest (4.34 ± 5.95 cm^2^) in the high threshold pressure range of bamboo sheet chair were smaller than the corresponding indexes of the office chair (81.430 ± 45.04 cm^2^, *p* = 0.00; 394.39 ± 98.99 cm^2^, *p* = 0.02; 13.54 ± 12.00 cm^2^, *p* = 0.00, respectively).

The pressure exponent, maximum pressure, and average pressure (2.68 ± 0.88 kPa, 6.66 ± 2.05 kPa, and 2.42 ± 0.59 kPa, respectively) of the bamboo sheet chair’s backrest were significantly lower than the corresponding indexes of the office chair (4.32 ± 1.62 kPa, *p* = 0.00; 10.50 ± 3.88 kPa, *p* = 0.00; 3.43 ± 0.97 kPa, *p* = 0.00, respectively). The maximum pressure and average pressure (26.26 ± 4.2 kPa, and 7.77 ± 1.30 kPa, respectively) of the seat pan were significantly lower than the corresponding indexes of the wooden chair (28.91 ± 4.90kPa, *p* = 0.00; 8.33 ± 1.41 kPa, *p* = 0.00, respectively).

Comfort evaluations for the three different chairs are listed in Table 4. Except for stability and lateral thigh pressure, most of the indices showed significant differences. The office and bamboo chair evaluation results were similar in most indexes, but there was a significant difference between their results and those of the wooden chair. The elastic feeling of the bamboo chair (2.28 ± 0.81) was lower than the wooden chair (4.76 ± 0.43, *p* = 0.00), and even lower than the office chair (2.76 ± 0.66, *p* = 0.01). The overall comfort index of the bamboo sheet chair (2.20 ± 0.49) was also lower than both the office (2.76 ± 0.99, *p* = 0.02) and wooden chairs (4.56 ± 0.64, *p* = 0.00).

### Subject characteristic parameters and pressure index

**Figure 5:**
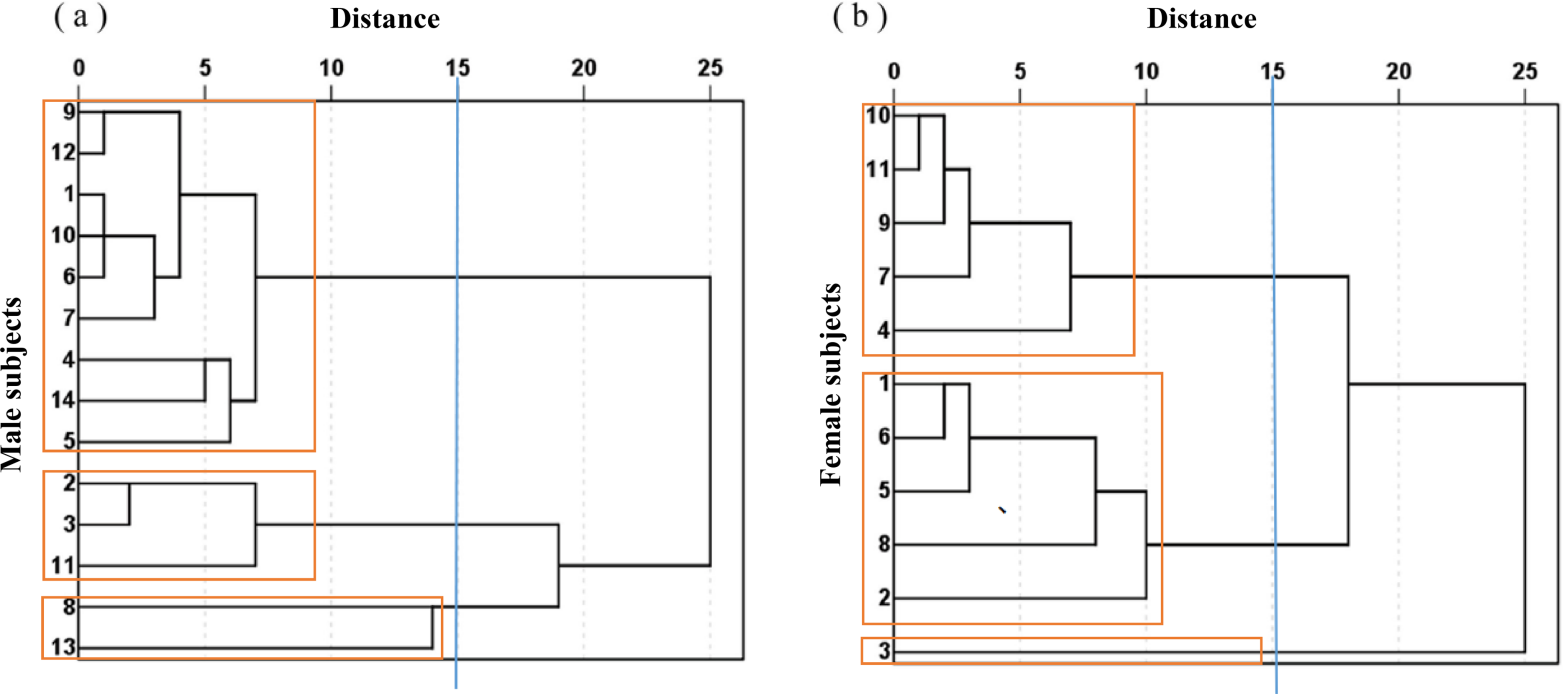
Cluster analysis tree diagram of subjects. (A) Cluster analysis tree diagram of male subjects; (B) cluster analysis tree diagram of female subjects.

**Figure 6:**
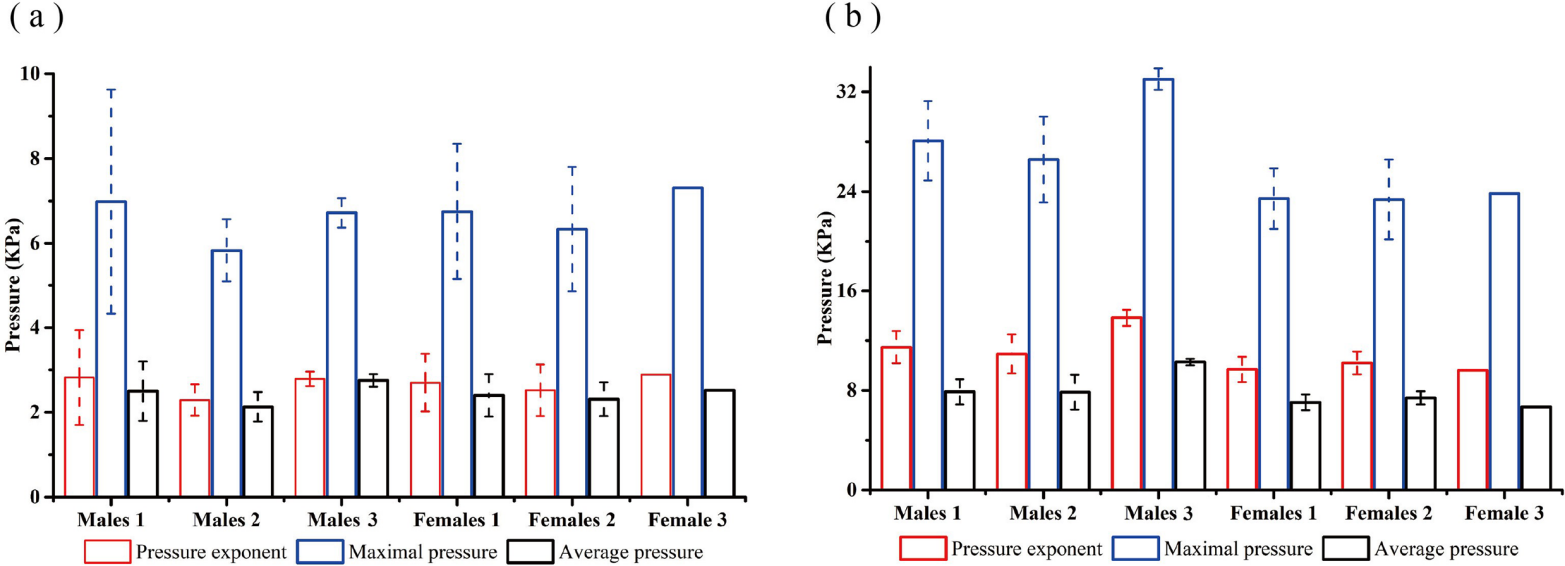
(A) The pressure index of human-chair backrest interface; (B) the pressure index of human-chair seat pan interface.

Figure 5 shows the cluster analysis used to divide the 14 male subjects into three groups and the 11 female subjects into three groups according to their body characteristics, represented by male groups 1, 2, 3 and female groups 1, 2, 3, respectively. The mean value and standard deviation for each group is listed in Table 5. Significant differences existed among most of the physical characteristic groups. In order to further analyze the relationship between the bamboo sheet chair and body characteristics, Table 6 lists the total contact area and the contact areas in low, middle-to-high and high-pressure threshold ranges. The human-chair interface pressure exponents, maximum pressure, and average pressure are shown in Fig. 6.

**Table 5:** The human body characteristic parameters (Mean + SD).

Index	Males 1	Males 2	Males 3	Females 1	Females 2	Female 3	*F*	Sig
Stature (cm)	171.43 ± 3.47	180.1 ± 2.47	169.45 ± 2.75	160.52 ± 2.68	160.94 ± 1.73	171.80	26.41	0.00
Weight (kg)	61.26 ± 3.70	73.80 ± 5.44	79 ± 7.77	50.66 ± 3.01	55.36 ± 2.10	52.50	22.92	0.00
Shoulder breadth (cm)	41.79 ± 1.58	44.86 ± 1.77	46.4 ± 2.54	39.78 ± 1.47	40.38 ± 1.73	40.40	8.19	0.00
Hip breadth (cm)	33.18 ± 2.16	34.63 ± 0.93	41.45 ± 2.45	33.54 ± 1.76	36.82 ± 1.69	34.50	3.81	0.02
Waist width (cm)	24.08 ± 3.21	26.00 ± 1.08	26.8 ± 0.14	20.9 ± 0.82	25.30 ± 0.84	21.60	3.65	0.02
The ratio of shoulder to hip	1.27 ± 0.09	1.30 ± 0.06	1.13 ± 0.10	1.16 ± 0.06	1.14 ± 0.07	1.17	2.67	0.05
BMI (body mass index)	20.87 ± 1.63	22.766 ± 1.88	27.48 ± 1.81	19.66 ± 1.08	22.12 ± 1.39	17.79	9.66	0.00
Sitting height (cm)	41.93 ± 2.86	41.83 ± 1.32	41.5 ± 1.40	33.68 ± 2.59	36.12 ± 0.83	38.40	3.24	0.03
Buttocks-popliteal length (cm)	44.02 ± 2.48	48.56 ± 1.79	43.30 ± 2.54	44.72 ± 2.05	45.1 ± 1.79	48.90	2.65	0.06
Hip thickness (cm)	19.45 ± 2.08	21.60 ± 2.29	19.85 ± 1.48	18.64 ± 1.59	23.36 ± 0.41	18.70	0.24	0.94

**Table 6:** The contact area on the human-chair interface:backrest/seat (Mean + SD).

Subject	Backrest				Seat			
	St/cm^2^	Sl/cm^2^	Sm/cm^2^	Sh/cm^2^	St/cm^2^	Sl/cm^2^	Sm/cm^2^	Sh/cm^2^
Males 1	307.70 ± 86.11	214.13 ± 62.22	52.08 ± 36.31	6.51 ± 9.20	802.77 ± 94.46	319.03 ± 36.31	249.58 ± 69.15	209.07 ± 48.01
Males 2	366.06 ± 62.01	284.31 ± 44.84	41.23 ± 36.12	2.17 ± 1.23	975.92 ± 116.72	332.78 ± 94.01	368.95 ± 75.21	250.31 ± 105.10
Males 3	404.76 ± 1.53	278.88 ± 23.01	96.57 ± 26.08	0 ± 0	948.42 ± 220.98	243.07 ± 98.21	316.86 ± 131.97	375.46 ± 190.29
Females 1	304.71 ± 63.52	213.99 ± 35.35	53.82 ± 42.76	4.34 ± 2.65	878.11 ± 103.04	340.30 ± 45.30	302.97 ± 82.18	204.44 ± 31.68
Females 2	305.58 ± 74.25	227.01 ± 58.59	42.10 ± 16.78	3.47 ± 4.50	964.92 ± 131.76	334.22 ± 41.32	360.70 ± 108.83	246.11 ± 40.03
Female 3	410.19	312.52	58.59	4.34	954.94	379.80	388.48	167.11

The results presented in Table 6 show that the backrest *St* and *Sl* for female subjects decreased with shorter statures and buttocks-popliteal lengths, while the seat pan *St* decreased with lower weight and hip breadths. The seat pan *Sh* for female group 2 (246.11 cm^2^) was larger than that of female group 1 (204.44 cm^2^, *p* = 0.02) and female group 3 (167.11 cm^2^, *p* = 0.00). The backrest *Sh* of male group 3 was 0 cm^2^, while the seat pan *Sh* was 376.46 cm^2^, which was larger than that of the male group 1 (209.07 cm^2^, *P* = 0.00) and male group 2 (250.31 cm^2^, *P* = 0.00).

For subjects with similar weights (differences within 2 kg), such as those in female groups 1 and 3, the seat pan pressure exponent and average pressure decreased with taller statures and longer buttocks-popliteal lengths, while the backrest pressure exponent and average pressure increased with greater statures and buttocks-popliteal lengths. The average seat pan pressure was greater than 4 kPa for all groups, and the average seat pan pressure for male group 3 was greater than 9.33 kPa.

## Discussion

In testing the pressure indexes of three different chairs, we found that the bamboo sheet chair was better at relieving pressure than the office and wooden chairs. The results presented in Table 2 show that the backrest and seat pan *Sh* of the bamboo sheet chair were smaller than those of the office chair. Previous studies have shown that increased capillary tissue interface pressures (TIPs) can cause blood flow occlusion, which leads to inadequate tissue oxygenation and subsequent tissue damage (Steinmetz & Langemo, 1997; Thosar et al., 2015). The pressure exponent, maximum pressure, and average pressure of the bamboo sheet chair’s backrest were lower than those of the office and wooden chairs, indicating that the back can get more uniform and soft support from the bamboo sheet chair. This demonstrates the mechanical properties of bamboo and the structure, pressure relieving effect, and adequate support of the chair’s design. The bamboo sheet chair’s seat pan pressure exponent, maximum pressure, and average pressure were also smaller than those of the wooden chair. Additionally, since the office chair’s seat pan and backrest are covered with a foam material, sitting in such chairs for long periods of time will produce a fuggy feeling (Zhang, Chen & Wu, 2018).

Figure 4 and Table 5 show that a shorter stature and buttocks-popliteal length gradually move the seat pan’s center of pressure distribution backwards, compressing the subjects’ coccyges. A shorter stature of subject (BMI differences within 2) causes part of the subject’s back to be suspended or located on the seat pan, and the back contact area begins to decrease. Relevant studies have shown that the human ischial tuberosity is strong and can withstand greater pressure (Newson & Rolfe, 1982), while the front of the inner thigh is rich in nerves and capillaries (Agliano et al., 1997). Excessive pressure is not conducive to blood circulation and nerve conduction (Keller, 2006). Therefore, a seat pan’s surface design should follow the principle of non-uniformity, gradually reducing pressure around the ischial tuberosity. A chair’s backrest is also very important in supporting the waist (Keegan, 1953). Lumbar suspension or excessive kyphosis are harmful to the spine (Ni et al., 2010).

Song, Zhang & Wang (2012) studied the effect of seat size parameter changes on body pressure distribution. The changes of maximum pressure, average pressure, contact area of seat pan and backrest surface, as well as the vertical pressure distribution curve in relation to seat height were obtained and the most comfortable sitting height of chair was finally obtained. This research provided some reference of sitting height for chair design. Makhsous et al. (2012) tested five chair designs on 15 young, healthy females. Sitting interface pressure and buttock-thigh tissue perfusion were measured during 10-min sitting on each chair. The research found that chair design significantly affected the distribution of the sitting pressure and buttock-thigh tissue perfusion. The research investigated the effect of five chair designs on interface pressure distribution and tissue perfusion in the buttock-thigh region, but the influence of different body characteristics on pressure distribution and sitting comfort was not discussed. BMI and body weight affect the body pressure distribution at the human-chair interface. Generally, a smaller BMI indicates a person is thinner with a more obvious ischial tuberosity and a more concentrated pressure distribution at the human-chair interface (Shi et al., 2019). For example, an increase in female subjects’ BMI causes the body pressure distribution at the human-chair interface to become more uniform. Figure 4 shows that the pressure at the coccyx was highest in female group 3, followed by female group 1, and female group 2 was the lowest. Higher BMI index means higher amounts of adipose tissue of subject, which is conducive to the dispersion of human-chair interface pressure.

From the data analysis in Tables 5–6 and Fig. 6, we concluded that:

(1) For female subjects, the backrest *St* and *Sl* increased with greater stature and buttocks-popliteal lengths, which was primarily due to the seat pan being suspended or located on the backrest. For female group 2, the backrest *Sh* was smaller than that of female groups 1 and 3, while the seat pan *Sh* was larger than those of female groups 1 and 3. This was mainly due to the fact that the shoulder and waist widths of female group 2 were larger than those of other two groups. Furthermore, female group 2’s back area was larger than those of female groups 1 and 3. It is worth noting that a large contact area is better at dispersing pressure.

(2) For male subjects, the backrest *Sh* of male group 3 was 0 cm^2^, while the seat pan *Sh* was 376.46 cm^2^, which were much larger than those of the other male groups. This was a result of their shoulder to hip ratio, popliteal height, and buttocks-popliteal lengths being the smallest. This resulted in pressure being concentrated on the seat pan, indicating that the backrest of the chair did not provide adequate back support. The thigh contact area is within the high-pressure threshold and is too large, which is not conducive to the blood circulation.

(3) We found that the seat pan’s pressure exponent and average pressure in female group 2 were the largest, not only because female group 2 had the largest body weight, but also because female group 2 had the smallest shoulder-to-hip ratio. Human body weight is mainly distributed in the lower half of the body (Shi et al., 2019). Although the lower body weight of female group 2 was higher, its maximum pressure was the smallest of the three groups. This is due to adipose tissue effectively dispersing the seat pan pressure of the human-chair interface.

(4) For subjects with similar weights (weight differences within 2 kg), such as female groups 1 and 3, the seat pan’s pressure exponent and average pressure decreased with shorter stature and buttocks-popliteal lengths, and increased with greater stature and buttocks-popliteal length. This is because the stature and buttocks-popliteal length of female group 3 were higher than those of female group 1, causing adequate contact with the seat backrest. A longer buttocks-popliteal length means that the subjects and backrest more fully come into contact, and the body’s weight is better distributed on the backrest of chair. However, when stature or buttocks-popliteal length is too high, it causes the backrest to put high pressure on the back of the human body.

(5) In both male and female groups, the maximum pressure on the seat pan was greater than 9.33 kPa, and the average pressure was greater than 4 kPa. The seat pan average pressure for male group 3 was even greater than 9.33 kPa, indicating that the chair was not suitable for overweight people to sit in for long periods of time because their muscles and soft tissues can be injured due to hypoxia (Elizebeth et al., 2015). The average back pressure of both male and female subjects was less than 4 kPa, indicating that the backrest interface was in a low-pressure condition.

## Conclusions

This work tested body pressure distribution of different subjects and found that people with larger trunk weights and thinner lower limbs tend to have excessive pressure concentrated on the seat pan and backrest, causing greater maximum pressure. People with longer stature or buttocks-popliteal lengths can more fully lean on the backrest, and the backrest can better share the weight of the human body. However, when stature or buttocks-popliteal length is too large, it can cause the backrest to put high pressure on the back of human body.

Higher amounts of adipose tissue and larger contact areas can effectively disperse the pressure of the human-chair interface, whereas a smaller BMI can concentrate pressure at the ischial tuberosity. A seat pan’s design should follow the principle of non-uniformity, which indicates that pressure should be gradually reduced around the ischial tuberosity. The bamboo sheet chair relieves pressure better than and similarly to wooden chairs and office chairs, respectively. The proper chair design, structure, and material can optimize the seat pan and backrest pressure distributions, and can greatly improve seat comfort.

##  Supplemental Information

10.7717/peerj.9476/supp-1 Data S1 Pressure distribution data

## Additional Information and Declarations

### Data Availability

The following information was supplied regarding data availability: The raw measurements are available as Supplemental File.

## References

[ref-1] Agliano M, Sacchi G, Weber E, Pucci AM, Comparini L (1997). Vasa vasorum of superficial collecting lymphatics of human thigh. Lymphology.

[ref-2] Aota Y, Iizuka H, Ishige Y, Mochida T, Yoshihisa T, Uesugi M, Saito T (2007). Effectiveness of a lumbar support continuous passive motion device in the prevention of low back pain during prolonged sitting. Spine.

[ref-3] Bi X, Wu Q, Zhao Y, Zhou H (2013). Research on long-term office chair comfort based on posture transform.

[ref-4] Blaga A (1984). Effect of the environment on the durability of plastic- based materials. Matériaux et Constructions.

[ref-5] Bui TH, Pradon D, Lestriez P, Debray K, Taiar R, Guillon B (2017). Influence of different types of wheelchair cushions for pressure ulcers in view of the experimental approach.

[ref-6] Chang CL, Hsieh MH (2016). Application of co-creation design experiences to the development of green furniture.

[ref-7] Chen YX, Shen LM, Guo Y (2010). Influence of seat height of the upholstered stool on sitting comfort. Journal of Anhui Agricultural University.

[ref-8] Chen YX, Shen LM, Guo Y, Liu SQ (2009). Influence of seat depth on comfortability based on body pressure distribution. Journal of Northwest Forestry University.

[ref-9] Collins M, Raleigh SM (2009). Genetic risk factors for musculoskeletal soft tissue injuries. Medicine and Sport Science.

[ref-10] Cristóbal JRS (2007). Effects on the economy of a decrease in forest resources: an international comparison. Forest Policy & Economics.

[ref-11] Dainoff M, Mark L, Ye L, Petrovic M (2007). Forget about aesthetics in chair design: ergonomics should provide the basis for comfort.

[ref-12] Elizebeth T, Sudhaya V, Silvia M, Singh SM (2015). A study of the factors associated with risk for development of pressure ulcers: a longitudinal analysis. Indian Journal of Dermatology.

[ref-13] Flander KD, Rovers R (2009). One laminated bamboo-frame house per hectare per year. Construction & Building Materials.

[ref-14] Forciea MA, Humphrey LL, Qaseem A (2015). Risk assessment and prevention of pressure ulcers. Annals of Internal Medicine.

[ref-15] Guo Y, Sheng LM, Chen YX (2011). Effect of the properties of sofa sponge backrest cushions on sitting comfort. Journal of Northwest Forestry University.

[ref-16] Hakeem KR, Ibrahim S, Ibrahim FH, Tombuloglu H (2015). Bamboo biomass: various studies and potential applications for value-added products. Agricultural biomass based potential materials.

[ref-17] Helander MG, Zhang L (1997). Field studies of comfort and discomfort in sitting. Ergonomics.

[ref-18] Horman I, Hajdarević S, Martinović S, Vukas N (2010). Numerical analysis of stress and strain in a wooden chair. Drvna Industrija.

[ref-19] Hu H, Yao Y, Ling L, Ran L, Zhao C, Xin Z, Rui W (2017). Research on pressure comfort of sofa based on body pressure distribution and subjective experience. International conference on digital human modeling and applications in health, safety, ergonomics and risk management.

[ref-20] Huang GM, Liu LL (2013). Study on low-carbon corrugated chair design. Applied Mechanics and Materials.

[ref-21] Kamp I (2012). The influence of car-seat design on its character experience. Applied Ergonomics.

[ref-22] Keegan JJ (1953). Alterations of the lumbar curve related to posture and seating. JBJS.

[ref-23] Keller BPJA (2006). Risks and risk-analysis for the development of pressure ulcers in surgical patients. Doctoral dissertation.

[ref-24] Li L, Shen LM, Zhou M (2009). Influence of sponge thickness on mattress comfort based on body pressure distribution. Journal of Northwest Forestry University.

[ref-25] Li NP, Zeng D, Wang Q, Long J (2010). Experimental study on the heat-transfer characteristics of new bamboo structure building. Science & Technology Review.

[ref-26] Liu C, Griffin MJ (2018). Measuring vibration-induced variations in pressures between the human body and a seat. International Journal of Industrial Ergonomics.

[ref-27] Lynch BM, Neville O (2015). Too much sitting and chronic disease risk: steps to move the science forward. Annals of Internal Medicine.

[ref-28] Ma G, Tian W, Sun H (2010). Research on chair design based on human dimensions’ application. Journal of Shenyang Jianzhu University (Natural Science).

[ref-29] Makhsous M, Lin F, Kruger SL, Lamantia A (2012). The effect of chair designs on sitting pressure distribution and tissue perfusion. Human Factors.

[ref-30] Miguel LT, Rosa P, José S, Tomás R (2008). Objective firmness, average pressure and subjective perception in mattresses for the elderly. Applied Ergonomics.

[ref-31] Newson TP, Rolfe P (1982). Skin surface po2 and blood flow measurements over the ischial tuberosity. Archives of Physical Medicine & Rehabilitation.

[ref-32] Ni HJ, Zhu XD, He SS, Yang CW, Wang CF, Wu DJ, Xu J, Li M (2010). An increased kyphosis of the thoracolumbar junction is correlated to more axial vertebral rotation in thoracolumbar/lumbar adolescent idiopathic scoliosis. Spine.

[ref-33] North EJ, Halden RU (2013). Plastics and environmental health: the road ahead. Reviews on Environmental Health.

[ref-34] Osorio L, Trujillo E, Van Vuure A, Lens F, Ivens J, Verpoest I (2010). The relationship between the bamboo fibre microstructure and mechanical properties.

[ref-35] Owen N, Sugiyama T, Eakin EE, Gardiner PA, Tremblay MS, Sallis JF (2011). Adults’ sedentary behavior: determinants and interventions. American Journal of Preventive Medicine.

[ref-36] Pheasant S (2005). Bodyspace: anthropometry, ergonomics and the design of work, 3rd edition. Journal of Economic History.

[ref-37] Salmon J, Tremblay MS, Marshall SJ, Hume C (2011). Health risks, correlates, and interventions to reduce sedentary behavior in young people. American Journal of Preventive Medicine.

[ref-38] Samuelsson K, Bjork M, Erdugan AM, Hansson AK, Rustner B (2009). The effect of shaped wheelchair cushion and lumbar supports on under-seat pressure, comfort, and pelvic rotation. Disability & Rehabilitation: Assistive Technology.

[ref-39] Scurlock JMO, Dayton DC, Hames B (2000). Bamboo: an overlooked biomass resource?. Biomass & Bioenergy.

[ref-40] Shelton F, Barnett R, Meyer E (1999). Full-body interface pressure testing as a method for performance evaluation of clinical support surfaces. Applied Ergonomics.

[ref-41] Shi YJ, Yuan FC, Chen T, Zhang TT, Guo Y, Chen YX (2019). The relationship between human body characteristic parameters and sofa functional dimensions. Journal of Anhui Agricultural University.

[ref-42] Song HY, Zhang JG, Wang F (2012). Influence of sitting height on human body pressure distribution. Journal of Tianjin University of Science & Technology.

[ref-43] Steinmetz JA, Langemo DK (1997). Changes in occipital capillary perfusion pressures during coronary artery bypass graft surgery. Advances in Wound Care: the Journal for Prevention and Healing.

[ref-44] Thosar SS, Bielko SL, Mather KJ, Johnston JD, Wallace J (2015). Effect of prolonged sitting and breaks in sitting time on endothelial function. Medicine & Science in Sports & Exercise.

[ref-45] Uysal M, Haviarova E, Eckelman CA (2015). A comparison of the cyclic durability, ease of disassembly, repair, and reuse of parts of wooden chair frames. Materials & Design.

[ref-46] Vink P, Hallbeck S (2012). Editorial: comfort and discomfort studies demonstrate the need for a new model. Applied Ergonomics.

[ref-47] Vlaović Z, Grbac I, Gojak I, Sekovanić I (2012). The influence of participants’ sex, mass, height and body mass index on pressures and comfort while sitting on office chairs.

[ref-48] Vollowitz E (1988). Furniture prescription for the conservative management of low-back pain. Topics in Acute Care and Trauma Rehabilitation.

[ref-49] Wang MG, Tian Y (2015). Furniture of environmental protection materials. Applied Mechanics and Materials.

[ref-50] World Medical Association (2013). World medical association declaration of Helsinki. Ethical principles for medical research involving human subjects. Journal of the American Medical Association.

[ref-51] Yang YM, Hui CM (2010). China’s bamboo culture/resources/cultivation/utilization. Technical Report—International Network for Bamboo and Rattan (INBAR).

[ref-52] Zhang XX, Chen MY, Wu HL (2018). Performance analysis of warp knitted spacer fabric in medical nursing pad. Journal of Xi’an Polytechnic University.

[ref-53] Zhang YH, Feng GQ (2015). China’s timber supply and demand: status and trend. Forestry Economics.

[ref-54] Zhang L, Helander MG, Drury CG (1996). Identifying factors of comfort and discomfort in sitting. Human Factors: The Journal of the Human Factors and Ergonomics Society.

[ref-55] Zhang JL, Jiang Y, Wang LY (2004). Multi-variable comfort index fuzzy evaluation instrument. Journal of Harbin Institute of Technology.

[ref-56] Zhang W, Zhang X (2011). The evolution of materials and processing technology in design of modern chair. Packaging Engineering.

[ref-57] Zhong ZY, Sun DL (2014). Analysis of chair’s materials and the wooden chair manufacturing process. Applied Mechanics and Materials.

